# COR-KNOT-induced aortic root injury during minimally invasive aortic valve replacement of a bicuspid aortic valve: a case report

**DOI:** 10.1186/s44215-025-00217-2

**Published:** 2025-07-24

**Authors:** Takahiro Ishigaki, Kazuma Okamoto, Satoshi Asada, Genichi Sakaguchi

**Affiliations:** 1 Department of Cardiovascular Surgery, KKR Sapporo Medical Center, Hokkaido, Japan; 2 Department of Cardiovascular Surgery, Kindai University Faculty of Medicine, Osaka, Japan; 3 Department of Surgery 1, Division of Cardiovascular Surgery, Hamamatsu University School of Medicine, Shizuoka, Japan

**Keywords:** COR-KNOT, Complication, Minimally invasive aortic valve repair, Aortic injury

## Abstract

**Background:**

The COR-KNOT device is widely used in minimally invasive cardiac surgery, and its efficacy in reducing operative time is well established. However, it has been associated with complications, such as valve leaflet perforation and prosthetic valve damage.

**Case presentation:**

We report a rare case of aortic root injury caused by COR-KNOT usage. A 72-year-old man with severe aortic stenosis due to a bicuspid aortic valve and distorted aortic root anatomy underwent minimally invasive aortic valve replacement using the COR-KNOT. After aortotomy closure, vigorous bleeding from the aortic root was observed. Punctate injuries of the aortic root, corresponding to the COR-KNOT clip sites, were identified following re-aortic cross-clamping. The procedure was converted to median sternotomy, the aortic root injury was repaired with an autologous pericardial patch, and the bioprosthetic valve was re-implanted using hand-tied sutures. The patient recovered uneventfully.

**Conclusions:**

This case highlights the importance of careful device selection and clip positioning in anatomically challenging cases to avoid life-threatening complications.

## Background

The COR-KNOT (LSI Solutions, Victor, New York, USA) is a device that enables reliable suture fastening even in deep and narrow surgical fields and is indispensable in minimally invasive cardiac surgery. However, it is associated with complications such as leaflet perforation, prosthetic valve injury, and delayed metallic embolization [1–3]. Serious complications such as aortic injury have rarely been documented. We report a case of aortic root injury associated with the use of the COR-KNOT during minimally invasive aortic valve replacement for a patient with a bicuspid aortic valve. This case highlights the importance of considering potential risk factors associated with the use of the COR-KNOT for anatomically challenging cases.

## Case presentation

**Fig. 1:**
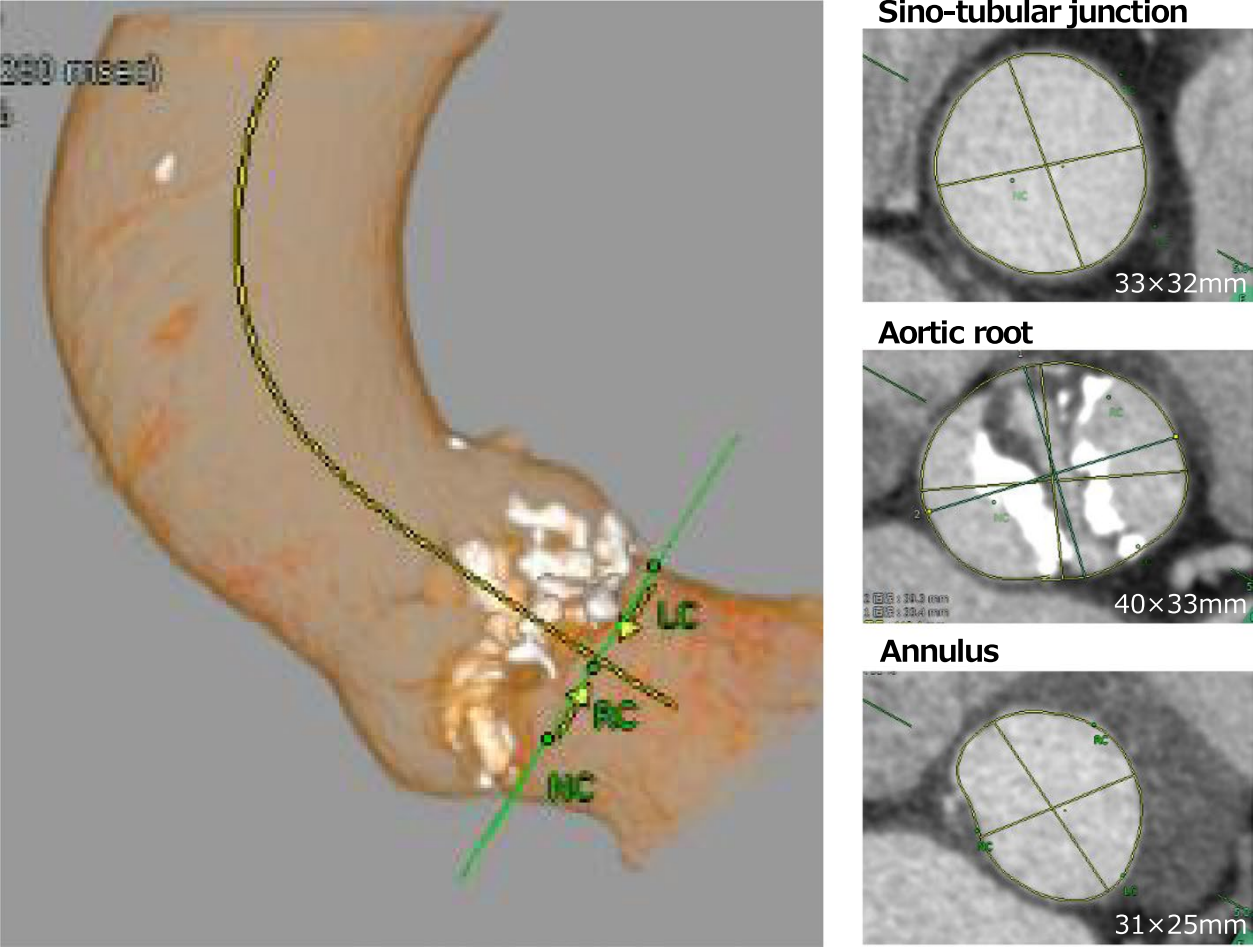
Preoperative computed tomography evaluation

A 72-year-old male patient presented with exertional dyspnea. His medical history included pleural plaques secondary to asbestos exposure and hypertension. Transthoracic echocardiography revealed a bicuspid aortic valve with fusion of the right and non-coronary cusps and severe aortic stenosis (valve area, 0.73 cm^2^; peak velocity, 4.7 m/s; mean gradient, 59 mmHg) without aortic insufficiency. The left ventricular function was preserved (left ventricular ejection fraction, 67%), and computed tomography revealed a 31 mm × 25 mm aortic annulus. The aortic root was distorted (40 mm × 33 mm), and the sino-tubular junction was 33 mm × 32 mm (Fig. 1).

**Fig. 2:**
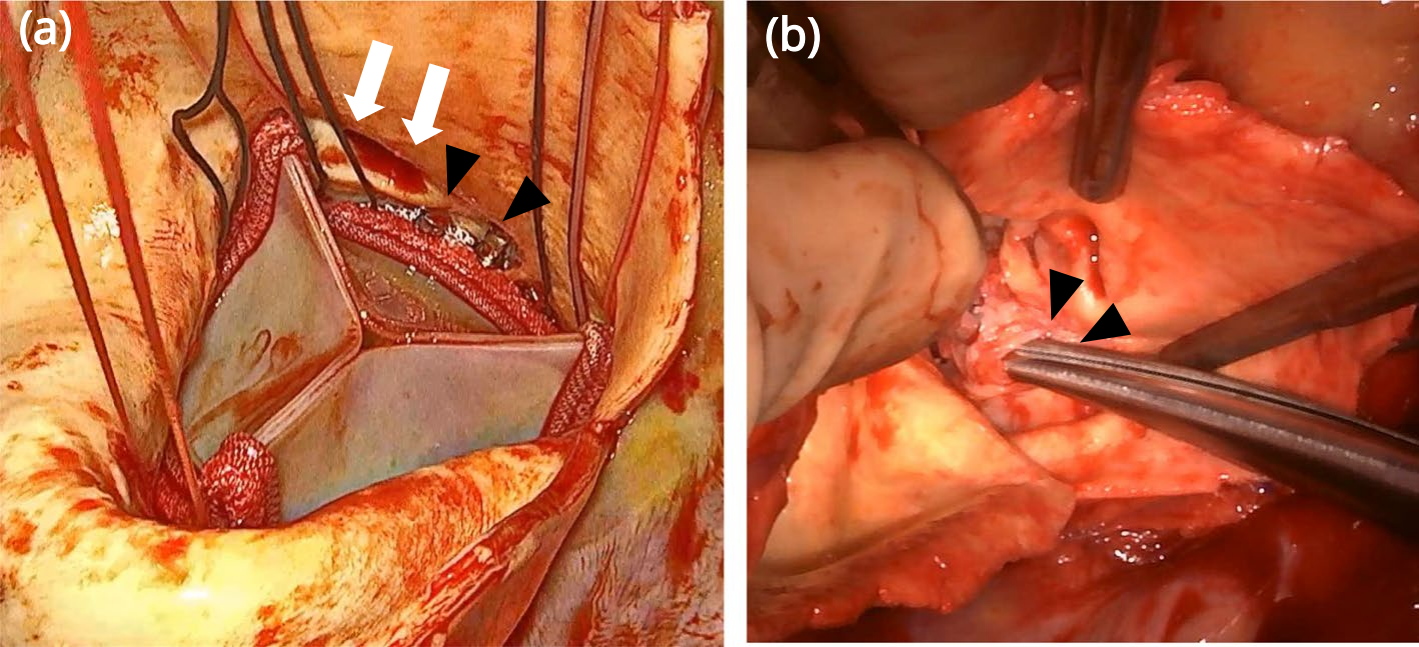
Interoperative findings with the use of the COR-KNOT device after prosthetic valve replacement (**a**) after implantation of the prosthetic valve. The tips of two COR-KNOTs (black arrowhead) are attached to the aortic root wall below the ostium of the right coronary artery (white arrow), **b** after removal of the prosthetic valve; injuries to the aortic root wall (black arrowhead) are observed where the metal clips are located

The patient underwent minimally invasive aortic valve replacement via right thoracotomy. Cardiopulmonary bypass was established with femoral artery and venous cannulation. After aortic cross-clamping and aortotomy, the calcified cusps were excised, and decalcification of the annulus was performed using a Cavitron ultrasonic surgical aspirator (Integra Life Sciences Corporation, Princeton, NJ, USA). A 25-mm Inspiris Resilia bioprosthesis (Edwards Lifesciences Corporation, Irvine, CA, USA) was implanted in the supra-annular position using the COR-KNOT. All COR-KNOT metal clips were implanted in the direction of the outside of the prosthetic valve. After aortotomy closure and aortic de-clamping, vigorous bleeding from the anterior aspect of the aortic root was observed. Re-aortic cross-clamping was performed, and punctate injuries of the aortic root were observed below the right coronary ostium, corresponding to the location of the metal clips (Fig. 2). Therefore, the procedure was converted to median sternotomy and the prosthetic valve was removed. The defect in the aortic root wall was repaired with an autologous pericardial patch. The same bioprosthetic valve was implanted again and hand-tied, and the aortotomy was closed. Postoperative transesophageal echocardiography confirmed the absence of aortic root pseudoaneurysms and paravalvular leakage. The patient recovered uneventfully and was discharged 20 days postoperatively.

## Discussion and conclusions

The COR-KNOT can shorten aortic cross-clamp and cardiopulmonary bypass times, especially effective in cases with concomitant procedures. However, it is important to note that the COR-KNOT can injure the leaflets of the native mitral valve in mitral valve repair using ring and aortic prosthetic valve cusps in aortic valve replacement, and such cases have been well-documented [1–3]. These complications are likely induced by mechanical irritation caused by the rigid metal clip. Therefore, positioning the metal clip in the direction of the outside of the prosthetic valve or ring is recommended. Conversely, the risk of aortic injury is considered low. To our knowledge, only one prior case has been described in the literature [4], and it was suggested that intimal damage can be caused by misalignment of the distal shaft of the COR-KNOT and its non-perpendicularity to the sewing ring. In the present case, the metal clip of the COR-KNOT may have contributed to aortic root injury, which was successfully repaired using autologous pericardium. However, if the injury had been more extensive or had progressed to aortic dissection, root replacement may have been necessary.

Sievers type 0 bicuspid aortic valves have a more elliptical annulus and distorted aortic root compared to that of normal tricuspid aortic valves or other types of bicuspid aortic valves [5, 6]. Implanting a circular prosthetic valve in an elliptical annulus may increase the distortion of the aortic root, potentially creating regions where the aortic root wall is in close proximity to the metal clip. Therefore, when aortic root deformation is significant, as often seen in the bicuspid aortic valve, the potential risk of aortic root injury associated with the use of the COR-KNOT exists [7, 8]. For these cases, the following strategies may be effective: (1) selecting a prosthetic valve with a sewing cuff positioned further away from the leaflets (such as the Epic porcine valve; St. Jude Medical, St. Paul, MN, USA) and placing the COR-KNOT in the direction of the prosthetic valve; and (2) avoiding the use of the COR-KNOT in areas where the distance to the aortic root wall is narrow and performing manual tying using a knot pusher. These techniques may help mitigate the risk of complications for patients with similar high-risk cases. Individual planning and technical modifications are essential when using the COR-KNOT in anatomically complex cases.

## Data Availability

Data sharing is not applicable to this article as no datasets were generated or analyzed during the current study.
